# History of Monte Carlo modeling of light transport in tissues using mcml.c

**DOI:** 10.1117/1.JBO.27.8.083002

**Published:** 2022-07-19

**Authors:** Steven L. Jacques

**Affiliations:** University of Washington, Department of Bioengineering, Seattle, Washington, United States

## Abstract

The Monte Carlo simulation called mcml.c was written and shared on-line since 1992. This perspective summarizes the contributions by the people involved in the development of mcml.c, and work by others extending the code.

The JBO Special Section Celebrating 30 Years of Open Source Monte Carlo Codes in Biomedical Optics (Volume 27 Issue 8) commemorates the open source versions of Monte Carlo modeling for biological tissues. About 30 years ago (1992) as the internet was just becoming popular, a program called mcml.c (Monte Carlo multi-layered) was posted on a website at the University of Texas M. D. Anderson Cancer Center (UTMDACC). After the year 2000, the program has been posted at the Oregon Medical Laser Center (https://omlc.org/software/mc), and has been widely used. The paper introducing the program (Wang et al.^1^) has been cited 4095 times as of 7 July 2022, according to Google Scholar. The mcml.c simulation has guided design of diagnostic and therapeutic devices and clinical protocols. This editorial recalls the people who participated in its development.

Steven Jacques in 1985 wrote an early Monte Carlo (MC) code while working as a research associate at the Wellman Center for Photomedicine, Massachusetts General Hospital, Boston. At that time, early developments of laser and light applications in medicine were being pursued. People were irradiating skin sites and examining biopsies to determine the damage or effect. However, the penetration of light into tissue was not well known, except for Kubelka–Munk simulations. Certainly, the computational methods were available in other fields (astrophysics, atmospherics, oceanics) but the medical laser community was still on a learning curve about computation. His MC simulation sampled an exponential probability for the photon stepsize.^2^ Jacques implemented a lookup-table approach to encoding various scattering functions and explored their effect on observable total and angular reflectance and transmittance for comparison with experiments on *ex vivo* skin.^3^ Steve is now an affiliate professor of bioengineering at the University of Washington, Seattle.

Marleen Keijzer wrote the second version of the MC code, working as a graduate student (Delft University, The Netherlands) in the Jacques lab. She wrote the steps of HOP, DROP, SPIN, CHECK in the current mcml.c that described the repeated actions on a simulated photon as it propagates until terminated. Her original names for these steps were in Dutch. This version was written in cylindrical coordinates, which proved to be an awkward approach, but she got it working. She implemented a model of aorta tissue (epithelium, mucosa, submucosa) and demonstrated the delivery of light into a multi-layered tissue.^4^ She demonstrated the delivery of excitation light into aorta and the collection of fluorescence from the tissue.^5^ At the time, there was a unique shape to the measured autofluorescence of aorta. Her work with another student, Rebecca Richards-Kortum in the MIT lab of Michael Feld, showed this shape to be strongly affected by reabsorption of autofluorescence by hemoglobin in the tissue. Marleen is a professor in the Delft Institute of Applied Mathematics of the Delft University of Technology (TU Delft), The Netherlands.

Scott Prahl wrote the third version of the MC code, as a graduate student (University of Texas-Austin in the lab of A. J. Welch).^6^ He collaborated in the Jacques lab on experimental goniometric measurements of light scattering (reflectance and transmittance) by *ex vivo* skin samples.^3^ These experiments showed scattering consistent with a Henyey–Greenstein (HG) scattering function, and he incorporated an analytic sampling of the HG function into the MC code.^7^ He simplified the code by using Cartesian coordinates.^8^ His work showed that HG scattering was a convenient and sufficiently accurate way to characterize and predict tissue scattering, and HG scattering has been widely used. In 1996 when the Jacques lab moved to the Oregon Medical Laser Center (OMLC) and the Oregon Health & Science University (OHSU) in Portland, Oregon, Scott also at OMLC created a widely used website for sharing the MC code (http://omlc.org/software/mc). Scott is a professor of electrical engineering and renewable energy (EERE) at the Oregon Institute of Technology (Oregon Tech).

Lihong Wang wrote in 1992 the fourth version of the MC code, as a postdoc in the Jacques lab at UTMDACC. He reorganized the code so that it handled multiple planar layers with each layer having unique optical properties including refractive index. The pre-compiled code used an input file to specify a particular simulation, which was very convenient and greatly helped many to use the code. He wrote the 173-page manual for the code (https://omlc.org/software/mc/man_mcml.pdf), in which he also verified the code by comparing to previous literature.^9^ He set up the first website at UTMDACC for sharing of the MC code. He was first author of the publication of the method, which is widely cited.^1^ Lihong is a professor of medical engineering and electrical engineering at the California Institute of Technology (Caltech).

In summary, this team of Steve, Marleen, Scott, and Lihong (Fig. 1) contributed to the mcml.c development. A key aspect of this effort was the commitment to an open source code and actively sharing the code on websites. Subsequently, improvements on mcml.c were made, as described next. However, mcml.c is simple to use and I often use mcml.c for solutions to problems.

**Fig. 1:**
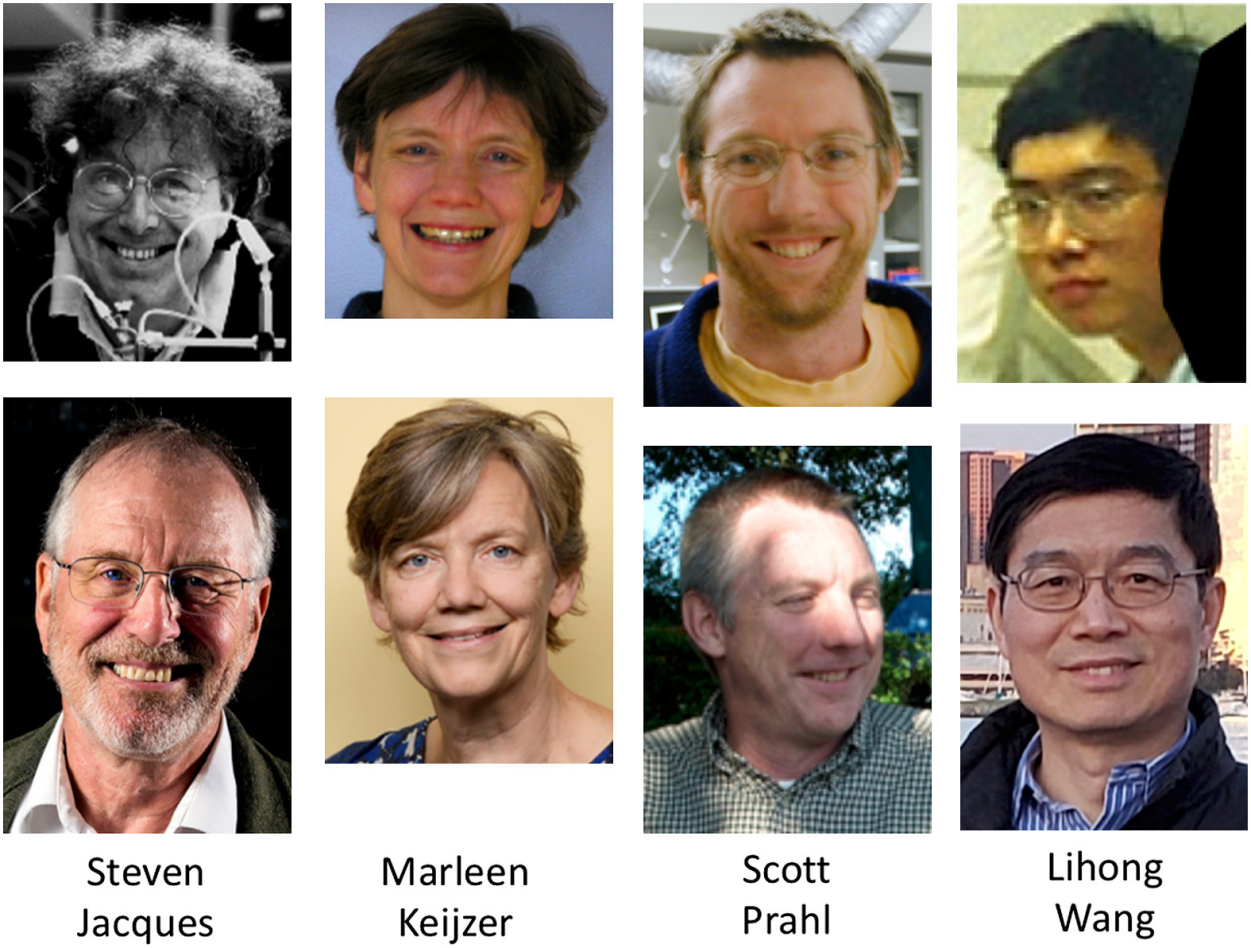
The team that built the Monte Carlo code in mcml.c.

Erik Alerstam et al. at Lund University in 2008 published a CUDA version of mcml.c to provide GPU-accelerated computations.^10^ This program is available for download (https://www.atomic.physics.lu.se/biophotonics/research/monte-carlo-simulations/gpu-monte-carlo), and greatly increases the speed of computation.

Jessica Ramella-Roman wrote a polarized-light version of mcml.c as a graduate student at OHSU in the Jacques lab (Fig. 2) collaborating with Scott Prahl.^11^^,^^12^ The code allowed simulations of the Stokes vector of escaping light (reflectance and transmittance) from a slab of tissue. Her code is publicly shared on the OMLC website (https://omlc.org/ software/polarization). Recently, a GPU-accelerated version of her code has been incorporated into MCX (Q. Fang, Northeastern University).^13^ Jessica is now a professor of biomedical engineering at Florida International University, Miami, Florida.

**Fig. 2:**
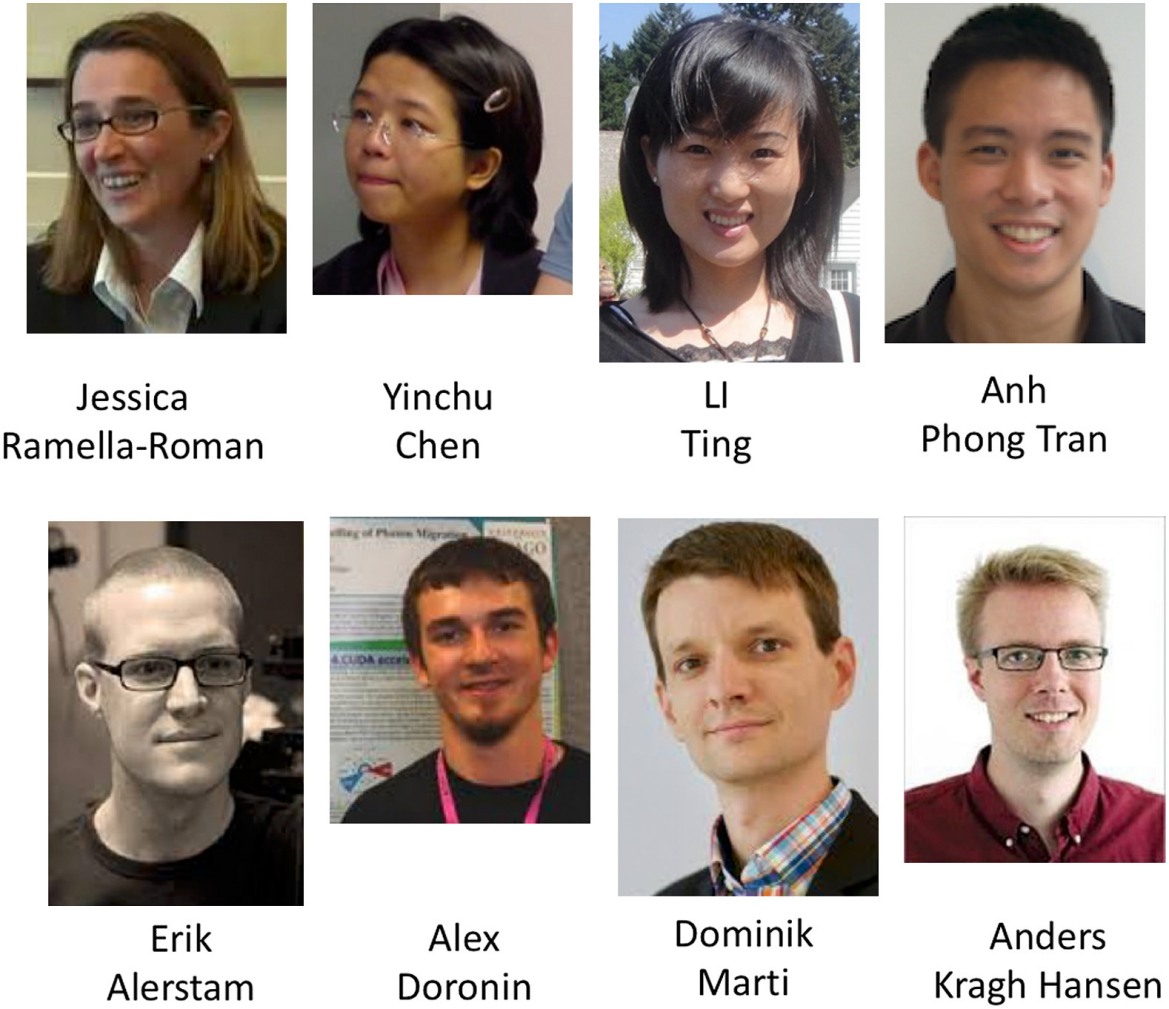
Others who modified mcml.c. Ramella-Roman implemented polarized-light MC code. Chen and Ting contributed to a voxelated version. Tran implemented a version that allowed 3D structures with varying refractive indices, where boundaries were smoothed between regions of differing refractive index. Alerstam implemented a GPU-accelerated version of mcml.c. Doronin implemented a GPU-accelerated version of mcml.c called mcxyz.c. Marti and Kragh Hansen developed a parallel processing version of mcxyz.c implemented within MATLAB as a teaching tool, called mcmatlab.

Yin-chu Chen as a graduate student at OHSU and OMLC in the Prahl lab (Fig. 2), implemented a voxelated MC code in 2005.^14^ Li Ting as a postdoc at OHSU in the Jacques lab (Fig. 2) implemented a voxelated version of mcml.c called mcxyz.c in 2014, inspired by the voxelated MC code of Boas et al.^15^ These two implementations were combined into the current version of mcxyz.c (https://omlc.org/software/mc/mcxyz). The program allows each voxel to be a different tissue type with unique optical properties.

Anh Phong Tran as a graduate student at Northeastern University, Boston (Fig. 2), modified mcxyz.c to allow for unique refractive indices (n) for each voxel. The program has a pre-processing step which finds smooth approximate boundaries between regions of voxels with shared n, such that refraction (reflectance and transmittance) at boundaries is based on the smooth boundaries and not on the cubic voxels themselves.^16^

Alex Doronin as a postdoc at Otago University, New Zealand, in Igor Meglinski’s lab (Fig. 2), implemented a CUDA version of mcxyz.c for GPU-accelerated simulations and shares the code on his website (https://github.com/aledoronin). Alex is an assistant professor in the School of Engineering and Computer Science at Victoria University of Wellington.

Dominik Marti, Anders Kragh Hansen, et al. (Fig. 2) at Denmark Technical University, Copenhagen, collaborated on a MATLAB-based version of mcxyz.c called mcmatlab to introduce students to MC modeling.^17^ The code is downloadable (https://gitlab.gbar.dtu.dk/biophotonics/MCmatlab), and runs from within a MATLAB environment. The code runs parallel processors so it is about 8-fold faster than mcxyz.c on a single CPU. It is an excellent vehicle for introducing students to Monte Carlo simulations.

There are many others who have implemented MC code in various forms for various purposes. This editorial does not attempt a review of MC simulations, but rather recounts the work that has built the original mcml.c code and some immediate improvements. The original goal of mcml.c was to provide an open source description of the basic kernel of computation for MC simulations so as to help others advance the programming. To be commemorating mcml.c after 30 years indicates some success toward that goal.
